# Epigenetics and Testicular Cancer: Bridging the Gap Between Fundamental Biology and Patient Care

**DOI:** 10.3389/fcell.2022.861995

**Published:** 2022-04-08

**Authors:** Alina-Teodora Nicu, Cosmin Medar, Mariana Carmen Chifiriuc, Gratiela Gradisteanu Pircalabioru, Liliana Burlibasa

**Affiliations:** ^1^ Faculty of Biology, University of Bucharest, Bucharest, Romania; ^2^ Department of Genetics, University of Bucharest, Bucharest, Romania; ^3^ University of Medicine and Pharmacy “Carol Davila”, Clinical Hospital “Prof. dr Theodor Burghele”, Bucharest, Romania; ^4^ Research Institute of University of Bucharest (ICUB), Bucharest, Romania; ^5^ Academy of Romanian Scientists, Bucharest, Romania; ^6^ Romanian Academy, Bucharest, Romania

**Keywords:** testicular germ cell tumor, DNA methylation, histone modifications, bivalent marks, miRNA, piRNA, epigenetic biomarkers, epigenetic-based therapy

## Abstract

Testicular cancer is the most common solid tumor affecting young males. Most testicular cancers are testicular germ cell tumors (TGCTs), which are divided into seminomas (SGCTs) and non-seminomatous testicular germ cell tumors (NSGCTs). During their development, primordial germ cells (PGCs) undergo epigenetic modifications and any disturbances in their pattern might lead to cancer development. The present study provides a comprehensive review of the epigenetic mechanisms–DNA methylation, histone post-translational modifications, bivalent marks, non-coding RNA–associated with TGCT susceptibility, initiation, progression and response to chemotherapy. Another important purpose of this review is to highlight the recent investigations regarding the identification and development of epigenetic biomarkers as powerful tools for the diagnostic, prognostic and especially for epigenetic-based therapy.

## Introduction

Testicular cancer (TC) refers to a multitude of malignancies affecting males, of which testicular germ cell tumors (TGCTs) account for over 90–95% of them (Ghazarian et al., 2015). Despite the high rate of patients’ survivability, TGCTs are of great concern because of the male infertility consecutive to common treatments toxicity (Hamano et al., 2017). TGCT is the most common cancer and a major cause of death in young adults between 15 and 45 years (Shanmugalingam et al., 2013; Ghazarian et al., 2017; Gurney et al., 2019).

In recent years, epidemiological studies have shown the lowest incidence in African and Asian countries and a significant increase in the incidence of TGCTs across European countries, with higher rates reported in Norway, Denmark, and Switzerland (Shanmugalingam et al., 2013; Gurney et al., 2019). However, significant differences appear between similar countries such as Poland and Slovakia and even amongst distinct ethnicities in the same country, suggesting the involvement of genetic and environmental factors in the initiation and development of TGCT (Ghazarian et al., 2015; Gurney et al., 2019).

The etiological factors are largely unknown, although urogenital abnormalities have been suggested, particularly cryptorchidism. Various risk factors have been investigated, from potential risk alleles to exposure to toxic substances such as bisphenol A (Landero-Huerta et al., 2017). More recently, epigenetic aspects of TGCTs with a major focus on DNA methylation have been highlighted (Brait et al., 2012). However, an emerging field is that of non-coding RNAs, which have been linked to the regulation of genes involved in germ cell differentiation, and which may play a critical part in TGCT development, infertility and even treatment susceptibility (Singh et al., 2021a).

## An Overview of Testicular Germ Cell Tumors

TC comprises types of cancer such as sex cord-gonadal stromal tumors, secondary testicular tumors and TGCTs (Boccellino et al., 2017). The latter are mainly classified as seminomatous (SGCTs) and non-seminomatous tumors (NSGCTs), and a third type, called spermatocytic tumors, accounting for only 1–2% of all TGCTs. Additionally, mixed tumors which exhibit both SGCT and NSGCT characteristics are classified under NSGCT. While SGCTs are slightly more common than NSGCTs, the increase in TGCT incidence over the past years has been attributed to an increase in NSGCT rates. NSGCTs include embryonal carcinomas, teratoma, yolk sac tumors and choriocarcinomas. These types tend to be more aggressive since they can spread more easily in the early stages (Ghazarian et al., 2015; Greene et al., 2016; Singh et al., 2021b).

Both SGCTs and NSGCTs originate in a precursor lesion previously known as intratubular germ cell neoplasia (ITGCN), currently named germ cell neoplasia *in situ* (GCNIS) (Brait et al., 2012; Moch et al., 2016). The precursor lesion is thought to take place during embryonic development, when primordial germ cells (PGCs) migrate to the gonadal ridge and differentiate into gonocytes. The gonocytes are similar to atypical cells of GCNIS in terms of morphology and protein expression and they might be responsible for the GCNIS (Landero-Huerta et al., 2017). However, SGCTs resemble PGCs, while NSGCTs may have extra-embryonic tissue components or even somatic differentiation (Martinot et al., 2018). SGCTs and NSGCTs have different biological features, which may be explained by their distinct expression profiles. While both subtypes express OCT3/4 and NANOG (pluripotency-associated transcription factors), SGCTs express only SOX2 and NSGCTs solely SOX17 (Buljubašić et al., 2018). At cytogenetic level, SGCTs tend to be hypotriploid whereas NSGCTs tend to be hypertriploid (Lafin et al., 2019).

Clinically, three main stages of the disease have been identified. Initially, the tumor is confined to the testicle, later on the tumor spreads to retroperitoneal lymph nodes, and finally the tumor spreads to other lymph nodes and organs (Smith et al., 2018). The first clinical stage tends to be more commonly identified in patients over 50 years of age, as opposed to younger patients (Dieckmann et al., 2018). On average, SGCT patients are older than NSGCT patients and disease seems to be less aggressive in their case. NSGCTs are more likely to form metastases, which may account for differences in later disease development (Oosterhuis and Looijenga, 2005; Dieckmann et al., 2018). The serum levels of classical tumor markers beta-human chorionic gonadotropin (bHCG), alpha-fetoprotein (AFP) and lactate dehydrogenase (LDH) have been shown to be significantly higher in NSGCTs than in SGCTs. Particularly, AFP could be a specific marker for NSGCTs since detection of abnormal levels in SGCT patients is scarce (Dieckmann et al., 2018; Laguna et al., 2019; de Vries et al., 2020). Overall, the two subtypes of TGCT may have the same embryonic origin, but their evolution diverges substantially, probably due to various molecular events which may be involved in defining the evolution of the initial lesion, as well as its prognosis (Umbreit et al., 2020).

## TGCT Risk Factors

Although the etiology of TGCTs is still unknown, various risk factors have been investigated, from genetic to microenvironmental aspects. The microenvironment of male germ cell development is complex and the impact of numerous contributors has been suggested, namely cryptorchidism, hypospadias, bleeding during pregnancy, estrogen exposure during pregnancy, high maternal age, premature birth, low birth weight (Singh et al., 2021a). However, the interplay between the genetic and environmental factors typical to cancers is likely the explanation for TGCTs, this being dubbed “the genvironmental hypothesis” (Baroni et al., 2019).

### Genetic Factors

The contribution of genetic factors to the development of TC seems to be one of the highest among all cancers, in spite of the low percentage of patients with familial cases. Nonetheless, having an affected brother or father greatly increases the risk of TC and the heritability of TGCT has been estimated at 37–49% (Litchfield et al., 2015a; Loveday et al., 2017). No particular genetic alteration has been pinpointed as a strong enough candidate for inducing TGCTs, however the highest correlation seems to be with single nucleotide polymorphisms (SNPs) within the kit-ligand gene (*KITLG*) (Litchfield et al., 2015b). Interestingly, *KITLG* is located on chromosome 12, whose amplification is frequently identified in TGCTs (Barrett et al., 2019). Alterations of the KITLG/KIT system have been associated with infertility in mice and increased risk of TGCT (de Vries et al., 2020). Additional genes located on this chromosome have been linked to tumorigenesis, namely Cyclin D2 (*CCDN2*) and V-Ki-ras2 Kirsten rat sarcoma viral oncogene homolog (*KRAS*) (Hacioglu et al., 2017; Ding et al., 2019).

Other risk loci that may correlate to TGCTs contain genes involved in microtubule assembly, telomerase function, DNA damage repair and epigenetic processes (de Vries et al., 2020). Such is the case of PRDM14, a DNA binding-protein which plays an essential role in establishing pluripotency in PGCs and embryonic stem cells (ESCs) by regulating DNA methylation (Seki, 2018). Moreover, PRDM14 suppresses the expression of previously mentioned genes *OCT4*, *NANOG,* and *SOX2* (de Vries et al., 2020).

Of particular interest is the androgen receptor (*AR*) gene, as hormone levels may play a role in TGCT development. Some polymorphisms of *AR* have been associated with an increased risk of TGCT and metastases, particularly two polymorphic regions within the first exon where trinucleotide repeats of CAG and CGC are present. However, the data is inconsistent (Martinot et al., 2018).

### Non-Genetic Anatomic, Hormonal and Environmental Factors

Despite their high heritability, TGCTs have a strong environmental component with recent studies suggesting that the increase in incidence observed mainly in industrialized countries could be attributed to harmful substances, for instance endocrine disruptors. Endocrine disruptors (EDs) are chemical compounds that alter endogenous steroid levels and implicitly their function by altering their synthesis, action, metabolism, elimination (Calaf et al., 2020). Several EDs are routinely used in industrialized countries and the level of potential toxicants to which humans are exposed daily is constantly rising. One of the most studied EDs is Bisphenol A (BPA), present in plastic objects subject to daily use. However, over the last years, its use has been regulated in several countries (Kadasala et al., 2016). BPA can bind to AR and interfere with its localization and function. Intriguingly, BPA has been shown to decrease the expression of estrogen-regulated miRNAs and increase levels of DNA methylation (Lombó and Herráez, 2021; Shi et al., 2021). Other sources of EDs could come from medical use, as is the case of diethylstilbestrol (DES), or from agriculture in the case of pesticides (Sharma et al., 2020). Similar to BPA, studies on female chicks have suggested that DES could alter miRNAs. The pesticide vinclozolin (VCZ) has also been reported to alter the expression of non-coding RNAs in the sperm of male rats descended from exposed females. The effect seems to have persisted over at least three generations (Lombó and Herráez, 2021).

Despite the relationship between previous exposure to EDs during organogenesis and urogenital abnormalities such as hypospadias, cryptorchidism and infertility, no clear link has been defined between EDs and TGCTs since initiating factors are difficult to determine, and the results obtained so far have been contradictory. For instance, polyvinyl chloride (PVC) and polychlorinated bisphenyls (PCBs) have been associated with an increased risk in some studies, but not in others (Vega et al., 2012). It is therefore important to note that these changes do not necessarily occur in PGCs, but in the Leydig and Sertoli somatic cells which are essential for spermatogenesis (Oosterhuis and Looijenga, 2019; Singh et al., 2021b). The EDs might apparently play a rather indirect role, considering that hormonal disturbances during pregnancy may increase the risk of TGCTs. Moreover, TGCTs are common in young males, including teenagers, which suggests that hormonal profile changes related to puberty may play an important part in transition from GCNIS to TGCT. At the same time, environmental factors can mediate epigenetic changes that could lead to TGCTs.

## Epigenetic Aspects in TGCT

Over the past decades, the field of epigenetics has come into spotlight, for it is able to fill in the blanks left by genetic studies. Epigenetic reprogramming is involved in normal male germline development and epigenetic changes are passed down through generations without any DNA sequence change (Oakes et al., 2007; Rousseaux et al., 2008; Ge et al., 2017). Questions regarding gene regulation, cytodifferentiation or chromosomal inactivation have now found answers in epigenetic mechanisms such as DNA methylation, histone modifications, chromatin remodeling that entails histone replacement with nuclear proteins involving incompletely elucidated mechanisms and RNA interference (Burlibaşa and Zarnescu, 2013).

Epigenetic reprogramming of the germline begins during embryonic development in a sex-specific manner. In males, PCGs migrate to the genital ridge in the sixth week of pregnancy where, together with somatic cells, form gonads capable of spermatogenesis. Spermatogenesis entails both mitotic and meiotic divisions and occurs continuously over the lifetime in a large number of cells, thus the processes need to be tightly regulated (Rousseaux et al., 2008).

Briefly, PGCs divide mitotically and differentiate into spermatogonia. Spermatogonia are divided in two types, type A, which replenishes the necessary stock of spermatogonia every cycle, and type B, which leads to primary spermatocytes. The latter divide meiotically leading to secondary spermatocytes and subsequently to spermatids. The spermatids undergo the process of spermiogenesis which results in spermatozoa formation. Over the course of these stages, several epigenetic modifications take place, which ensure proper functioning of germinal cells, as well as optimal post-fertilization development (Oakes et al., 2007; Ge et al., 2017). Above all, the maximum condensation capacity of DNA is reached through spermiogenesis via an unique form of chromatin remodeling that entails histone replacement with nuclear proteins. However, the mechanisms of this process are incompletely elucidated (Burlibaşa and Zarnescu, 2013).

During embryogenesis, global demethylation occurs in order to ensure totipotency; however, imprinted genes and repetitive sequences maintain their methylation status as this is critical for post-implanting development. *De novo* methylation takes place during the blastocyst stage, through the action of DNMT3a and DNMT3b, which reestablish methylation patterns, while DNMT1 maintains them. PCGs undergo reprogramming before migrating to the genital ridge, erasing all existent methylation patterns that later get reestablished (Oakes et al., 2007; Rousseaux et al., 2008; Rothbart and Strahl, 2014).

Another important epigenetic mechanism is histone modification. PGCs lose the H3K9me2 marker and gain H3K27me3 after the first week of development in mice (Kota and Feil, 2010). Several histone modifications take place during spermatogenesis, most notably a global acetylation of histone tails which serves as a facilitator for histone replacement. Firstly, histones are replaced with testis-specific variants, then with transition proteins and finally with protamines, this process leading to global transcriptional inactivation (Bao and Bedford, 2016). During meiosis, H3K4me1/2/3 and H3K9me2 markers suffer global distribution modifications (Kota and Feil, 2010). Moreover, the aberrant regulation of H3 modifications leads to germ cell apoptosis, infertility, and defective spermiogenesis (Yuen et al., 2014). Our previous studies have shown a unique pattern of H3 methylation in male germ cells, illustrating epigenetic crosstalk between H3K4me3 and DNA methylation during spermatogenesis (Burlibaşa et al., 2021).

With genetic factors hardly explaining the etiology of TGCTs and bearing no significance to the steady rise of cases over the years, recent studies have focused on the epigenetic factors which are exceptionally sensitive to environmental agents (Ghazarian et al., 2015). Epigenetic crosstalk may prove useful in the characterization of TGCTs, as isolated modifications do not generally play a big part in tumor initiation and development, but each contributes to the bigger picture. DNA methylation, histone modifications and non-coding RNAs are progressively more accepted as important mechanisms in tumor development, with studies only beginning to scratch the surface of their potential for diagnostic markers, therapeutic agents or targets.

### DNA Methylation

DNA methylation is notably the most studied epigenetic mechanism, having been investigated in a variety of organisms and settings for the past couple of decades. TGCTs make no exception, given that DNA methylation is recognized as being an important progression factor and may even be involved in the initiation of the tumor. The biggest concern coupling methylation and tumors is the hypermethylation of tumor suppressor genes, which leads to their inactivation (Costa et al., 2017). Additionally, the expression patterns of DNMTs support this idea, as DNMTs are mainly expressed in the fetal testis and in undifferentiated spermatogonia, which correlates to the GCNIS hypothesis (Oakes et al., 2007).

Expression patterns of DNMTs are highly regulated during spermatogenesis, with an increased expression being observed in spermatogonia, followed by a decrease in spermatocytes and subsequent cell types (Song et al., 2014). However, these patterns seem to be altered in TGCTs, with DNMT1, DNMT3a, DNMT3b, DNMT3l being overexpressed in embryonal carcinoma. Moreover, DNMT3b has been proposed as a marker for SGCTs relapse (Matsuoka et al., 2016; Costa et al., 2018; Laguna et al., 2019).

DNA methylation has been studied both at genome and gene levels, as it is a major event involved in germ cell development and differentiation. Different types of TGCTs have been associated with distinct genome-wide methylation patterns. For instance, a global hypomethylation is associated with SGCTs, GCNIS and gonadoblastoma (tumors which contain germ cell and sex cord/gonadal stroma), while hypermethylation is largely identified in NSGCTs, particularly in teratoma, yolk sac tumor, and choriocarcinoma (Landero-Huerta et al., 2017; Martinot et al., 2018; Laguna et al., 2019). The remaining NSGCT type, embryonal carcinoma, presents an intermediate level of methylation and non-CpG methylation resembling embryonic stem cells (Smiraglia et al., 2002).

A focus has been laid on the genes involved in pluripotency such as the previously mentioned *NANOG* gene, which is characterized by a hypomethylated state in spermatogonia, and hypermethylation in spermatozoa. In TGCTs, its promoter methylation level correlates to different states of differentiation. For instance, SGCTs and embryonal carcinoma, which contain undifferentiated cells that resemble PCGs or ESCs, are associated with high *NANOG* expression levels, while teratomas, yolk sac tumors and choriocarcinomas have low levels or do not express *NANOG* at all, as their cells are more differentiated (Vega et al., 2012; Buljubašić et al., 2018). Similarly, the *OCT3/4* gene has been found to be hypomethylated in SGCTs and embryonal carcinoma, which indicates faulty pluripotency suppression that could have been responsible for tumor development (Martinot et al., 2018). Table 1 shows the percentages of CpG methylation of several genes involved in NSGCTs and SGCTs.

**TABLE 1:** Methylation percentages of CpG islands promoter genes in SGCT and NSGCT, gene function and clinical significance of differential promoter methylation.

Gene	Function	Methylation percentage of gene promoter		Clinical significance of promoter methylation	References
		SGCT	NSGCT		
*RASSF1A* (Ras association domain –containing protein 5)	Tumor suppressive potential, role in cellular homeostasis	0–40	21–83	Biomarker for morphologically heterogenous tumors; prognostic value for NSGCTs; predictive biomarker of TGCTs response to cisplatin-based therapy	Koul et al. (2002)
					Koul et al. (2004)
					Markulin et al. (2017)
					Costa et al. (2018)
					Dubois et al. (2019)
					Raos et al. (2021)
*MGMT* (O^6^-alkylguanine DNA alkyltransferase)	Involved in genome stability, prevents mismatch and errors during DNA replication and transcription	0–24	20–69	Biomarker for NSGCTs diagnosis; predictive biomarker of TGCTs response to cisplatin-based therapy	Smith-Sørensen et al. (2002)
					Koul et al. (2002)
					Koul et al. (2004)
					Brait et al. (2012)
					Martinelli et al. (2017)
					Markulin et al. (2017)
					Costa et al. (2018)
*SCGB3A1* (Secretoglobin family 3A member 1)	Negative regulation of cell growth; regulation of cell proliferation; cytokine activity	0	54	Biomarker for NSGCTs diagnosis and prognostic; identification of more clinically aggressive tumors	Lind et al. (2006)
					Lind et al. (2007)
					Costa et al. (2018)
*BRCA1* (Breast cancer type 1 susceptibility protein)	Tumor suppressor involved in pathways important for DNA damage, double-strand break repair, transcription regulation and chromatin remodeling	0-26	0	Under investigation as biomarker for SGCTs	Koul et al. (2002)
					Koul et al. (2004)
					Friedenson, (2005)
					Chovanec and Cheng, (2019)
*HOXA9*	Homeotic gene that acts as a regulator of embryonic development; replicative immortality in testicular cancer	0	26	Biomarker for NSGCTs diagnosis	Lind et al. (2006)
					Costa et al. (2018)
					Brotto et al. (2020)
					Raos et al. (2020)
					Paço et al. (2020)
*APC* (Adenomatous polyposis coli)	Multifunction tumor suppressor	0	24–29	Biomarker for NSGCTs diagnosis	Koul et al. (2002)
					Koul et al. (2004)
					Tanwar et al. (2011)
					Markulin et al. (2017)
*FHIT* (Fragile histidine triad gene)	Tumor suppressor	0	6–29	Biomarker for NSGCTs diagnosis	Koul et al. (2002)
					Kraggerud et al. (2002)
					Koul et al. (2004)
					Markulin et al. (2017)
*HOXB5*	Homeotic gene that acts as a regulator of embryonic development; tumor suppressor	0	13	Under investigation for NSGCTs diagnosis	Lind et al. (2006)
					Paço et al. (2020)
*CDH13* (Cadherin-13 or T-cadherin)	Involved in regulation of cell growth, survival and proliferation	0–6	9–12	Under investigation for NSGCTs diagnosis	Lind et al. (2006)
					Elshimali et al. (2013)
*CDH1* (Epithelial cadherin or E-cadherin)	Tumor suppressor	0	4–11	Under investigation for NSGCTs diagnosis	Koul et al. (2002)
					Elshimali et al. (2013)
*RARB* (Retinoic acid receptor beta–RAR-beta)	Nuclear receptor. It binds retinoic acid which mediates cellular signaling in embryonic morphogenesis, cell growth and differentiation	0	5–6	Biomarker candidate for early detection of TGCTs; promising predictive biomarker of TGCTs response to cisplatine-based therapy	Koul et al. (2004)
					Honorio et al. (2003)
					Costa et al. (2018)
					Lobo et al. (2021)

NSGCTs subtypes present locus specific promoter methylation that is distinct for each histological subtype, with genes such as *HOXA9, SCGB3A1, MMP9, CSFR1, MGMT, RASSF1A* being abnormally methylated (la Rosa et al., 2017; Singh et al., 2021a)*.* Tumor suppressor genes such as *RASSF1A*, *BRCA1, APC, CDH1, FHIT* also have hypermethylated promoters in TGCTs compared to normal tissues, and have been presented as novel target genes hypermethylated at high frequencies among nonseminomas (Table 1) (Lind et al., 2007; la Rosa et al., 2017; Mirabello et al., 2012). Additionally, risk alleles such as *KITLG, PDE11A, SPRY4, BAK1* present increased promoter methylation in familial TGCT cases (Lawaetz and Almstrup, 2015; Landero-Huerta et al., 2017). Appropriately, deletion of *KITLG* in mice has been shown to result in an increased TGCT susceptibility (Haugen et al., 2019). On the other hand, Sprouty RTK Signaling Antagonist *(SPRY4*) knockdown mice have been associated with inhibited TGCT growth and metastasis, warranting further studies (Das et al., 2018). Sporadic cases have shown increased methylation of *APOLD1, RGAG1, PCDH10* (Landero-Huerta et al., 2017).

Of particular interest is also the methylation status of retrotransposons, as their silencing is essential to gametogenesis. SGCTs tend to have demethylated LINE1 and ALU elements, while NGSCTs present demethylated LINE1 elements, but partially methylated ALU elements (Okamoto, 2012; Singh et al., 2021b). DNA methylation may also be involved in mechanisms pertaining to drug resistance, since cisplatin treatment can increase DNA methylation, and hypermethylation of genes such as *MGMT, RASSF1A, CALCA* and *HIC1* have been associated with cisplatin resistance (Yerby, 2021).

Another aspect concerning DNA methylation involves the modification of the 5-methyl-cytosine (5mC) at the amine or the methyl group, generating different marks. For instance, 5-hydroxymethyl-cytosine (5hmC) is formed through the addition of a hydroxyl group to the methyl group and can be further modified to 5-hydroxymethyl-uracil (5hmU) or 5-formyl-cytosine (5-fC) (Moore et al., 2013). These products are involved in the active DNA demethylation pathways and in recent years a role in various cancer types has been suggested for 5hmC in particular, with low levels generally correlating with a poor prognosis (Storebjerg et al., 2018). As an intermediate product of cytosine modification, 5hmC could be a key of the epigenetic regulation process and a potential marker in correlation with different stages of cancer (Xu and Gao., 2020). A study by Munari et al. showed low levels of 5hmC in SGCTs and high levels in differentiated teratoma, embryonal and yolk sack tumors (Munari et al., 2016). Further studies are necessary, as observations on Sertoli cells have indicated a role in testicular development, but 5hmC studies have so far focused on cancer types other than TGCTs (Munari et al., 2016; Landfors et al., 2017).

### Histone Modifications and Bivalent Marks

Analysis of tumor cells highlighted an aberrant pattern of histone modifications at individual promoter gene level or globally. Thus, most studies have focused on aberrant histone modifications within an individual site or a single histone modification, rather than on targeting combined modifications or abnormalities of enzymatic activity. Therefore, this section aims to review the role of bivalent markers and some enzymes involved in testicular cancers.

Histones are subject to various post-translational modifications including methylation, acetylation, phosphorylation, ubiquitinilation, sumoylation, ADP-ribosylation, deamination, formylation, O-GlcNAcylation, propionylation, citrullination, butyrylation and the newest described, crotonylation (Kouzarides, 2007; Tan et al., 2011; Li et al., 2012). These modifications function in an orchestrated manner, a phenomenon described as “*the histone code*” that provides transcriptional plasticity and has an important role in long-term regulation of cell phenotype (Strahl and Allis, 2000). Schreiber and Bernstein (2002) have proposed a more general hypothesis where histone posttranslational modifications serve as a nuclear DNA-associated signal transduction pathway. The addition or removal of an array of covalent modifications in histones is catalyzed by the histone modifying enzymes.

Methylation and acetylation of histones, two of the most important histone modifications involved in the epigenetic regulation of genes, especially during embryonic development and differentiation, are mediated by specific enzymes. The table below summarizes the main enzymes involved in these changes (Table 2).

**TABLE 2:** Various classes and subtypes of enzymes that mediate histone methylation and acetylation.

Type of enzyme	Family/group	Enzyme	References
HMTs (PRMTs –arginine-methyl transferases)	Type I	PRMT 1, PRMT 3, PRMT 4/CRM1, PRMT-6, PRMT-8	Chen et al. (1999)
			Bedford, (2007)
	Type II	PRMT 5, PRMT 9/FBXO11	Branscombe et al. (2001)
	TYPE III	PRMT7	Branscombe et al. (2001)
HMTs (KMTs—lysine methyltransferases)	SET1 (SET domain)	EZH I, EZH2	Jenuwein, (2006)
	SET 2 (SET domain)	SMYD2, NSD1-3, SETD2	Schotta et al. (2008)
	SUV39 (SET domain)	SUV39H1, SUV39H2, G9a, ESET//SETDB1, GLP, CLLL8/SETDB2	Beck et al. (2012)
	RIZ (SET domain)	RIZ 1, BLIMP1/PRDM1, PFM1/CRS2	Wood and Shilatifard (2004)
			Irifuku et al. (2016)
			Wang et al. (2018)
	Seven-β-strands	Dot1/DOT1L	Okada et al. (2005)
HDMs	KDM1	KDM1A, KDM1B	Kaniskan et al. (2018)
	JMJC	KDM2-8	Hyun et al. (2017)
HATs	GNAT	KAT2A, KAT2B	Marmorstein and Trievel, (2009)
	p300/CBP	KAT3B	Marmorstein and Trievel, (2009)
	MYST	KAT7, KAT8, KAT5, KAT6A	Marmorstein and Trievel, (2009)
	Transcription coactivators	KAT4, KAT12	Wapenaar and Dekker, (2016)
	Steroid receptor	KAT13A, KAT13B	Wapenaar and Dekker, (2016)
	Cytoplasmic	HAT1, HAT4	Parthun et al. (1996)
HDACs	CLASS 1	HDAC1, HDAC2	Khan et al. (2008)
	CLASS II	HDAC4, HDAC5, HDAC6, HDAC10	Guardiola and Yao (2001)
			Gregoretti et al. (2004)
			Lombardi et al. (2011)
	CLASS III	Sirtuins (SIRT 1–7)	Dai and Faller, (2008)
	CLASS IV	HDAC 11	Haberland et al. (2009)

Many enzymes are involved in histone methylation with individual or multiple levels of methylation. The differences depend on the modified histone, targeted amino-acid sites and the number of methyl groups that are added to histones (Jambhekar et al., 2019). The functional disturbance of some chromatin modifying enzymes, as listed in Table 2, has been directly implicated in germ cell tumor initiation and progression. The elucidation of the molecular pathway of these enzymes could help us to mediate their function and to modulate gene expression in cancer treatment.

#### Histone Methylation in Testicular Cancer

**FIGURE 1:**
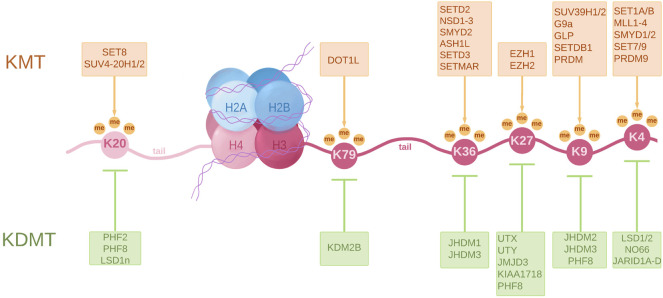
A schematic representation of a nucleosome showing principal lysine methylation sites on histones H3 and H4 and the associated lysine methyltransferases (KMT) and lysine demethylases (KDMT). KMTs transfer one to three methyl groups to specific lysine residues. These are associated with different functions such as transcriptional activation, commonly involving H3K4me2/3, H3K36me3 and H3K79me3, transcriptional repression in the case of H3K9me2/3, H3K27me2/3 and H4K20me3, and even DNA repair in the case of H4K20me2. KDMTs remove these methyl groups and help establish a tight regulation of gene activity. The full scope of histone methylation is, however, extremely complex as it involves a certain “histone code” that regulates the spatiotemporal differences in gene expression.

During normal development, histone modifications act together with transcription factors (TFs) and chromatin modifiers to control the spatio-temporal regulation of gene expression patterns. In this context, the identity of each cell type, its associated gene expression pattern and its epigenetic signature, is maintained and subsequently inherited by daughter cells (Sarmento et al., 2004; Dambacher et al., 2010). Alterations in the histone modification pattern occur in the early stages and accumulate during tumorigenesis. The main lysine methylation sites on histone H3 and H4 that are regulated by HMTs (Histone Methyltransferases) and HDMs (Histone Demethylases) are summarized in Figure 1.

**FIGURE 2:**
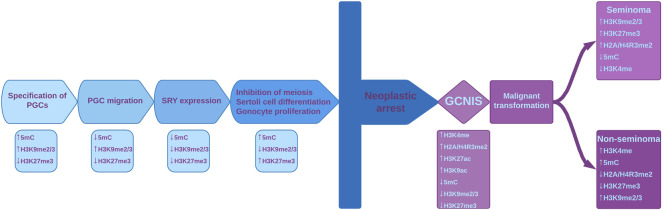
Epigenetic reprogramming in male gamete lineage and the epigenetic disturbances during malignant transformation. The main events that take place during normal development are shown, with their specific epigenetic modifications depicted under each event. Chromatin modifying enzymes play important regulatory roles during fetal gonadal development by regulating histone marks and DNA methylation. Histone marks and 5meC are important regulatory set points during normal fetal gonadal development but also in the neoplastic transformation in pathogenesis of TGCT. Due to various factors, neoplastic arrest can occur and normal development is thus halted. This leads to GCNIS which is associated with disturbances of epigenetic modifications. GCNIS has ESC-like features, including the presence of bivalent markers. At some point, seminomatous or non-seminomatous tumors can develop, with their listed specific modifications. PGCs–primordial germ cells; SRY–sex determining region Y protein; GCNIS - germ cell neoplasia *in situ*; ESC–Embryonic stem cell.

It is known that PCGs undergo epigenetic reprogramming (Seki, 2018). When PCGs begin their migration into genital ridge, they already contain genomic imprints, and starting with the genital ridge colonization, several epigenetic modifications occur, including the erasure of H3K9me2, associated with decreased levels of HP1 (heterochromatin protein 1). Additionally, a progressive increase in H3K27me3, a repressive mark mediated by the polycomb group protein EZH2 (Enhancer of zeste), and in active marks H3K4 methylation, respectively H3K9 acetylation, occurs (Figure 2) (Surani and Reik, 2006; Burlibaşa and Gavrilă, 2011).

H3K27me3 and H3K4me3 have antagonistic properties on chromatin conformation and function, but the simultaneous presence (bivalent domain) of these modifications is an elegant epigenetic feature originally identified in key differentiation genes within embryonic stem cells (ESCs), providing epigenetic plasticity and maintaining pluripotency. H3K4me3 prevents the permanent silencing of genes, perhaps by preventing DNA methylation, whereas H3K27me3 assures that gene expression levels remain low (Figure 2). As ESCs differentiate, bivalent loci lose one of the two histone marks and become fully activated or stably silenced (Kumar et al., 2021). Bivalent domains are frequently associated with CpG islands (Ku et al., 2008; Zaidi et al., 2017). KMT2 is a protein that contains a DNA binding domain involved in recognition of hypomethylated CpGs and may play a role in targeting the DNA methylation complex to bivalent domains (Allen et al., 2006). The complexity of the gene regulation process is reflected by the establishment and maintenance of the bivalent marks H3K4me3 and H3K27me3, as well as the sequential addition of other histone modifications and the completion of this histone code with DNA methylation (Strahl and Allis, 2000). These bivalent marks are important in long-term regulation and play key roles in regulating the balance between stem cell proliferation and differentiation (Zaidi et al., 2017). A study performed by Kumar et al. (2021) revealed that bivalency in ESCs does not poise genes for rapid activation but protects promoters from *de novo* DNA methylation challenging the idea that H3K4me3 at bivalent chromatin is a signal for rapid activation of transcription and the loss of H3K4me3 at CpG island make them more susceptible to aberrant DNA methylation during aging and cancer (Kumar et al., 2021).

During differentiation, switches in bivalent domains can control the expression of critical lineage-specific genes that gain or lose these modifications (Bernstein et al., 2006). Recently, bivalent marks have been found at the promoter of cancer related genes involved in the development of those tissues, suggesting a mechanism by which cancer cells reacquire some properties that are characteristic of undifferentiated, pluripotent cells (Jadhav et al., 2016). These genes are active during organogenesis, and an aberrant epigenetic modification or other factors can leave them with transcriptional potential in adult cells, leading to tumorigenesis (Jadhav et al., 2016; Terranova et al., 2021).

Scientific literature is poor in data regarding histone modifications in TGCTs. In a normal testis, histone H3 methylation has stage-specific distribution, while in non-seminoma, histone H3K4 and H3K9 methylation has been detected in all cellular stages (Lambrot et al., 2012; Vega et al., 2012; Burlibaşa et al., 2021). This suggests an association between methylation of histone H3K4/H3K9 and abnormal gene expression in non-seminoma. In GCNIS, low levels of repressive histone modifications H3K9me2 and H3K27me3 and high levels of H3K4 methylation have been detected (Almstrup et al., 2010; Facchini et al., 2018). Additionally, Eckert et al. suggest that dimethylation of H2A/H4R3 could be a mechanism by which seminomas and intratubular germ cell neoplasia maintain their undifferentiated state, while the loss of these histone modifications is observed in non-seminoma tumors concomitant with somatic differentiation (Eckert et al., 2008).

According to (Singh et al., 2021a) EC and TGCTs may have high levels of bivalent histone marks H3K27me3 and H3K4me3, similar to pluripotent ESCs and induced pluripotent stem cells. High levels of H3K4me3 protect chromatin from DNA methylation, an essential mark for sperm differentiation, leading to the maintenance of proliferative capacity of this tumoral cell type (Singh et al., 2021b).

Considering that individual modifications are associated with various testicular cancer subtypes, the enzymes responsible for the mentioned modifications are also the subject of studies aiming to elucidate the mechanisms behind tumor initiation, progression and development. For instance, Suv39h1 and Suv39h2 are lysine-methyl transferases that mediate histone H3 di- and/or trimethylation at lysine 9 (Schotta et al., 2008). Methyltransferases, such as SUV39 act as tumor suppressors and the low expression in tumor cells may result in increasing cell proliferation, apoptosis resistance and poor differentiation (Liu et al., 2018).

G9a (EHMT2) is a mono/dimethyltransferase of H3K9 essential for early embryonic development. G9a has been found in association with transcriptional repressors and contributes to transcriptional silencing. It is expressed in all somatic tissues and at high levels in the testis, where it plays a crucial role in germ cell development, being a target of retinoid signaling (Volle et al., 2009).

Another histone methyltransferase is EZH2, which is involved in trimethylation of histone H3K27 (Chang and Hung, 2012). EZH2 is found in round spermatids during spermatogenesis and it is involved in epigenetic reorganization leading to an extreme compaction of chromatin. In TGCTs reduced levels of EZH2 compared to normal testis have been detected (Lambrot et al., 2012).

LSD1/KDM1 is a histone lysine-demethylase that converts H3K4me2 to H3K4me or unmethylated H3K4, leading to suppression of gene expression. An increased level of LSD1 protein has been observed in various types of pluripotent cancer cells and in human testicular seminoma tissues (Karakaidos et al., 2019).

Starting with the Chromatin Immunoprecipitation Sequencing (ChIP–seq) assay optimization, which combines Next Generation Sequencing (NGS) with ChIP, a comprehensive analysis for the identification of transcription factors binding to histone modifications can be performed.

Furthermore, bivalent chromatin marks and their regulators have been identified as a target for cancer therapeutics because of the role in the maintenance of pluripotency. In this context, mathematical algorithms have been developed as well as an *in silico* platform able to identify the best combination of conventional and epigenetic drugs suitable for the treatment of those heterogeneous cancer cell populations with non-uniform response (Alarcón et al., 2021).

#### Histone Acetylation in Testicular Cancer

Histone acetylation is mediated by specific enzymes called histone acetyltransferases (HAT) and histonedeacetylase (HDAC). Acetylated forms of histones have been found during normal spermatogenesis.

Hyperacetylation of histone H4 is the most important modification during spermatogenesis (Dhar et al., 2012). This signal plays a crucial role in the replacement of histones with protamines, which is a key mechanism for nucleus condensation, differentiation of spermatozoa and fertility (Burlibaşa and Gavrilă, 2005; Burlibaşa and Zarnescu, 2013). There are very few studies in the scientific literature regarding the presence of this modification in testicular cancer, but existing ones indicate the absence of hyperacetylated H4 in all types of testicular cancer except GCNIS (Faure et al., 2003). Also, in GCNIS high levels of H3K9 acetylation along with the presence of hyperacetylated H4 have been detected (Almstrup et al., 2010; Facchini et al., 2018). Moreover, disturbances in histone acetylation could be involved in TGCTs initiation considering that a higher expression for all three HDACs isoforms from class I has been detected (Jostes et al., 2019).

An important challenge for the future research will be to understand the roles of changes in histone modification patterns together with chromatin modifying enzymes in cancer progression and metastasis. Deciphering the histone code in cancer cells can help us to predict, prevent and treat testicular cancers. Epigenetic drugs like histone modification inhibitors could be an alternative option for current TGCTs therapy.

### MicroRNAs and piRNAs in Testicular Cancer

The gene expression is one of the most important fundamental processes for all living organisms. In recent years, new mechanisms of gene expression regulation have been discovered.

MicroRNAs (miRNA) are small, single-stranded non-coding RNA involved in the regulation of post-transcriptional gene expression via translational repression, mRNA cleavage, and deadenylation (O’Brien et al., 2018). They contribute to crucial embryological functions, including organogenesis in normal development (Burlibaşa et al., 2008; Ahmad, 2016; Boland, 2017; Gross et al., 2017). They have also been implicated in tumorigenesis of various different solid organ and haematological malignancies (Conduit and Tran, 2021), infertility (Salas-Huetos et al., 2019; Barbu et al., 2021), drug resistance (Ma et al., 2010) and immune response (Mehta and Baltimore, 2016).

This class of RNAs is a group of molecules with an average of 22 nucleotides in length that do not code for proteins, but are able to bind the 3′untranslated regions (UTRs) of several transcripts, leading to the degradation of the targeted mRNAs and to the inhibition of translation. miRNAs can prevent the process of tumor formation and, in the case of aberrant expression, they could also promote it (Regouc et al., 2020).

Many miRNAs are known to originate in the introns of their pre-mRNA host genes. The majority of all currently identified miRNAs are intragenic and processed from introns and relatively few exons of protein coding genes, but there is a small part of miRNAs, which are intergenic, transcribed and regulated independently (de Rie et al., 2017). In some instances, miRNAs are transcribed as one long transcript called cluster.

Beyond involvement in the relevant biological processes, the alteration of the miRNA pathways in cancer cells has been used for diagnosis, prognosis and monitoring cancer treatment response. They are detectable and stable in blood and semen (Mitchell et al., 2008). Moreover, they can be released following cell death (Das et al., 2019) and thus, they can be used as potential circulating tumor markers.

In germ cell tumors, two main clusters of miRNA are overexpressed i.e. the *miR-371–373* cluster including: *miR371a-3p*, *miR-372a-3p*, *miR-373a-3p* and the *miR-302* cluster including: *miR-367*, *miR-302d*, *miR-302c-5p*, *miR-302c-3p, miR-302a-5p*, *miR-302a-3p*, *miR-302b-5p* and *miR-302b-3p*. In addition, other miRNA molecules are involved in the mechanisms of testicular tumorigenesis.

#### The *miR371-373* Cluster

The *miR-371*, *miR-372*, and *miR-373* are a group of miRNA genes located in 19q13.4 position (Rippe et al., 2010). Members of the *miR-371–373* cluster are the most abundant miRNAs in human embryonic stem cells. They are involved in self-renewal processes. Therefore, they have a major regulatory role in maintaining the pluripotency status of ESCs (Ghasemi et al., 2018).

This cluster includes a promising miRNA member used as a biomarker in testicular cancer diagnosis. Up-regulation of miR-371–373 has been found in TGCT patients (Gillis et al., 2007) and also in other types of cancers. *miR-371–373* cluster may activate the Wnt/β catenin mechanism, thus sustaining cell proliferation and invasion characteristics of cancer cells (Regouc et al., 2020).

Although numerous studies have highlighted the role of this cluster in the molecular mechanism of malignant transformation, only miR-371a-3p has been extensively analyzed as a marker for diagnosis, staging, and prognosis in TGCT, and has been reported as an accurate diagnostic tool able to discriminate between various testicular histotypes (Regouc et al., 2020). Furthermore, this marker seems to be the first discovered miRNA suitable for metastatic assessment and for monitoring cisplatin treatment in TGCTs patients (Regouc et al., 2020). A study performed by Vilela-Salgueiro et al. (2018) described the differential expression of miR-371a-3p in various testicular tumor types, highlighting that seminomas’ patients displayed the highest level of this marker, followed by embryonal carcinomas, teratomas and yolk sac tumors.

The miR-372 and miR-373 are biomarkers with a minor role. Differences in the expression pattern of miR-372 and miR-373 in TGCT and healthy testes have been found, but further studies are needed to use them as standardized tumor biomarkers (Wei et al., 2015; Murray et al., 2016).

#### The *miR-302* Cluster

The *miR-302/367* cluster was mapped in an intron on the 4q25 region of human chromosome 4, and transcribed in a long pri-miRNA, which is then processed in eight miRNAs: miR-367, 302d, 302c-5p, 302c-3p, 302a-5p, 302a-3p, 302b-5p, and 302b-3p (Gao et al., 2015). The *miR-302* cluster has a crucial role in regulation of the cell cycle in embryonic and pluripotent stem cells by interacting with cell cycle regulators. In addition, its members can target different epigenetic factors and signaling cascades such as the Akt/PKT pathway, promoting survival and growth in response to extracellular signals (Lin, 2011).

The *miR-302* members are involved in repression of lysine-specific histone demethylase 1 and 2 (AOF1 and AOF2) and methyl-CpG binding proteins (MECP1 and MECP2). This interaction leads to the destabilization of DNA methyltransferase 1, and promotes reprogramming and cells development (Lin et al., 2011).

A study performed by Das et al. (2019) indicates a possible role of *mi-R302* members in the development of TGCTs. The study demonstrates that a lower level of miR-302 downregulates the SPRY4 expression, which subsequently decreases cell growth. SPRY4 is a potential tumoral factor that may be overexpressed in TGCT and interferes with PI3K/Akt signaling pathway (Das et al., 2019). Even if these findings underscore the involvement of mi-R302 in TGCTs, more studies are needed to confirm its potential as a tumor marker.

The *miR-367* is located nearby to *miR-302* cluster and therefore they are often called the *miR-302/367* cluster. In a study performed by Syring et al. (2015), the serum level of miR-367-3p was significantly higher in TGCT patients compared to healthy males. Other studies emphasize the superiority of miR-371-3p in the prediction of viable tumor tissue after chemotherapy (Leão et al., 2018), while others propose miR-367-3p as a key indicator of chemotherapy-resistant disease and metastasis (Rosas Plaza et al., 2019). In conclusion, more studies are needed to answer whether the expression level of miR-367-3p will add value when it is supplemented with miR-371-3p.

#### Other miRNA

##### miR-517/519

The *miR-517a-3p*, *miR-519a-3p,* and *miR-519c-3p* are three miRNAs that belong to the same group (*C19MC*), located near the *miR-371–373* cluster on chromosome 19 (Bentwich et al., 2005). A study performed by Flor et al. (2016) demonstrates that the expression of this cluster’s members in stage I seminomas and teratoma mixed tumors is lower or identical to normal testes. On the contrary, high expression level was detected in stage III advanced tumors and non-seminomatous tumors. These findings sustain the role as a potential biomarker for advanced-stage tumor screening and histological type tumor identification (Flor et al., 2016).

##### miR-449

This miRNA has a major role in normal spermatogenesis and interferes with Cyclin Dependent Kinase 6 (CDK6) to regulate progression of the cell cycle. In testicular cancer, miR-449 expression is lower than in healthy testicular tissue (Yong-Ming et al., 2017; Conduit and Tran, 2021). Silencing of *miR-449* may occur in tumor cells, but more data are needed so that it can be considered a potential tumoral biomarker.

##### miR-383

miR-383 regulates cell cycle, proliferation and apoptosis in TGCTs (Tian et al., 2013). Its expression is high in embryonic carcinoma, binds to interferon regulatory factor -1 (IRF1), leading to its downregulation simultaneously with CDK2 and p21. Furthermore, high expression blocks the phosphorylation of H2AX, the major histone variant involved in DNA repair, causing an increased tumor’s sensitivity to treatment (cisplatin). In this context, miR-383 could have a potential target for treatment in embryonic carcinomas (Huang et al., 2014).

##### miR-223-3p

miR-223-3p play an important role in cell growth and apoptosis in various tumor types (Calin and Croce, 2006). miR-223-3p expression has been found higher in TGCTs than normal testes and has been shown to promote cell proliferation in TGCT cell lines (Liu et al., 2017). The target of miR-223-3p is F-box/WD repeat-containing protein 7 (FBXW7), a tumor suppressor factor. The overexpression of mir-223-3p correlates with a decrease in FBXW7 leading to the progression of TGCTs (Kanatsu-Shinohara et al., 2014).

##### miR-506/514 Cluster

*miR-514a-3p* is a member of *miR-506/514* cluster potentially involved in apoptosis. In embryonal carcinomas and seminomas, a lower expression has been detected. This miRNA interacts with paternally expressed gene 3 (*PEG3*) to activate *p53*. In TGCT, the overexpression of miR-514a-3p inhibits apoptotic mechanisms leading to tumor development and progression (Özata et al., 2017).

##### *Let-7a* and *miR-26a*

These two miRNAs have been found acting as a tumor suppressor in different human cancers (Conduit and Tran, 2021). By interaction with HMGA1 (High Mobility Group A), they inhibit seminoma cell growth. Let-7a and miR-26a have lower expression in human seminomas (De Martino et al., 2020).

**FIGURE 3:**
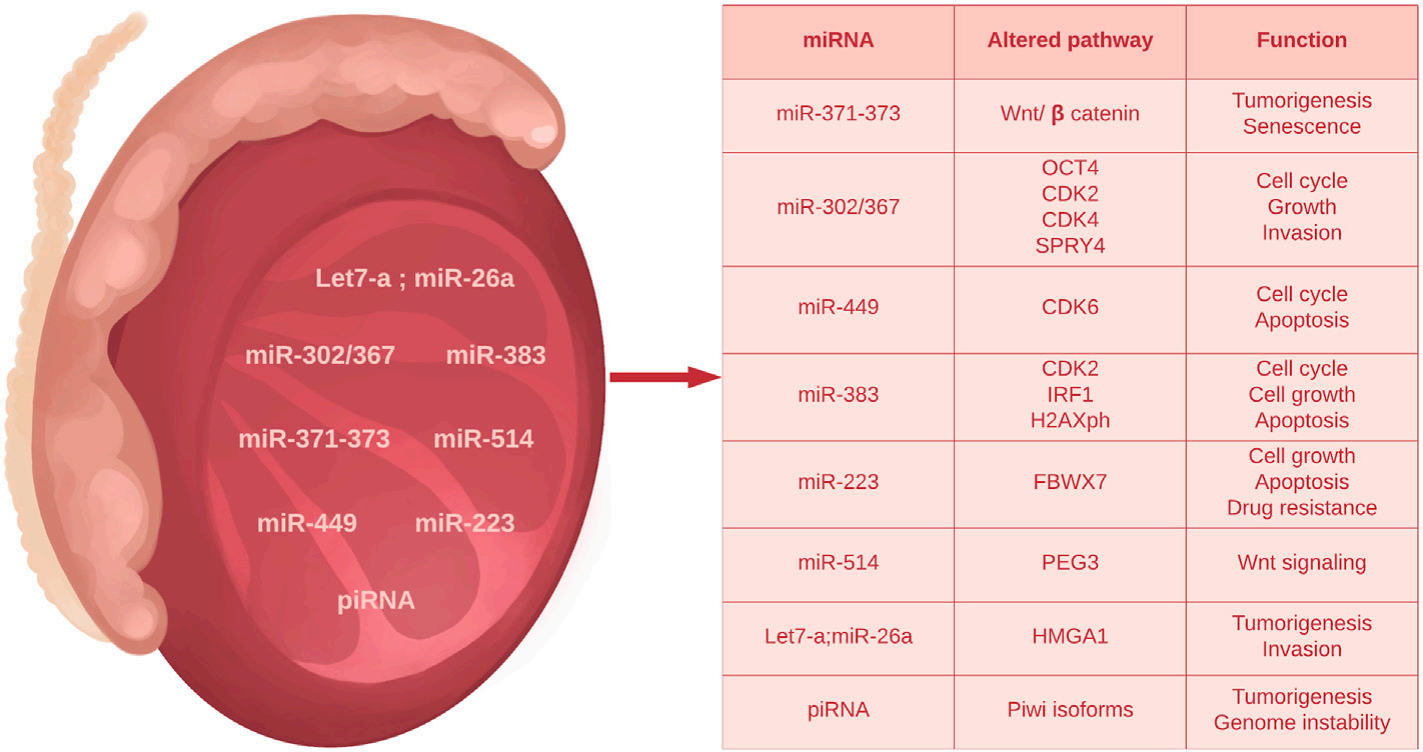
Summary of main microRNAs and piRNAs deregulated in TGCTs.

An overview of miRNAs and their potential interaction with tumor suppressive or oncogenic signaling pathways is depicted in Figure 3.

#### piRNAs

piRNAs (PIWI- interacting RNAs) are an intracellular class of small non-coding RNAs (24-30 nt in length) mainly expressed in germ cells lineage, that play an important role in epigenetic processes such as histone methylation and modification, silencing of transposons and maintenance of the sexual cells’ stability (Reuter et al., 2011). These small molecules are transcribed from genomic repetitive sequences and transposable elements.

The main pathway of piRNA mechanism is via PIWI interacting protein, a subfamily of Argonaute proteins. PIWI protein is normally expressed in germ line and by attachment to the piRNA it drives the main processes of genome integrity maintenance (cleavage of transposable element transcripts and heterochromatin formation associated with an increased level of DNA methylation) (Gainetdinov et al., 2018).

Compared to other non-coding RNAs, the role of piRNAs in cancer is less known, only a few studies on piRNA-PIWI mechanisms having been published. Gainetdinov and his coworkers (2018) examined the function of PIWI-piRNA in four stages of TGCTs development: germ cells from normal adult testes, germ cells adjacent to TGCTs, TGCT cells and CIS cells. They found that in normal testes and adjacent to TGCTs four isoforms of PIWI are expressed: PIWIL1, PIWIL2, PIWIL4 and TDRD1. They did not observe the expression of any of the isoforms listed above in the three cancer cell lines studied. Aberrant expression of PIWI orthologs in human TGCTs was first detected in seminomas (Goh et al., 2015).

The decreased expression of some piRNAs has been reported both in seminoma and non-seminoma tumors (Gainetdinov et al., 2018). In addition, aberrant hypermethylation and lower level of PIWI-family genes expression have been detected, alongside the downregulated piRNAs. Overall, these results reveal the existence of a cancer specific hypermethylation pattern in CpG islands, associated with piRNAs, which drives their transcriptional silencing in testicular cancer.

These findings suggest that the conventional germline-like PIWI/piRNA pathway vanishes during transition from germ cells to cancer cells (Gainetdinov et al., 2018). However, more data are needed to fully understand the role of the PIWI-piRNA complex in tumorigenesis and to validate it as a biomarker in testicular cancers.

## TGCT Therapy

TGCTs have an impressive 5-year survivability rate of approximately 95%, that, even in case of metastases, could reach 90% (Liao et al., 2018). Current treatment options include surgery, radiation therapy, and chemotherapy (Oing et al., 2018). Typically, a combination of different chemotherapy agents are used, such as bleomycin, etoposide and cisplatin (Siddiqui et al., 2020). From these therapeutic agents, cisplatin and bleomycin sensitivity is correlated with epigenetic processes. Cisplatin and bleomycin are representatives of DNA damaging agents, also named genotoxins. Cisplatin is a metal-based agent used for decades to treat patients of various types of cancer like cervical, ovarian, bladder, lung and TGCTs (Dasari and Tchounwou, 2014; Aldossary, 2019). TGCTs are particularly sensitive to this treatment, compared to other cancer types. SGCTs and ECs are the most chemosensitive, while teratomas are the most resistant, which draws a parallel to somatic-derived tumors being more resistant to treatment than TGCTs (Singh et al., 2019a). However, due to the fact that TGCTs most commonly develop in males 15–45 years of age, there is a particular concern for the fertility of the affected individuals and the health of their future offspring (Huyghe et al., 2004; Delbes et al., 2007). Besides drug-elicited toxicities, the emergence of chemoresistance is reported in around 10–15% of the tumors today (Fung et al., 2018; Groot et al., 2020). The patients developing resistance to treatment have a poor quality of life and eventually die within a few months, since there are no defined targeted treatments for these patients (Gómez-Ruiz et al., 2012; Allen et al., 2017; Hellesnes et al., 2021). Cisplatin resistance occurs by pre-target, on-target and post-target mechanisms. It has been suggested that TGCTs activate DNA damage-response mechanisms under the selective pressure induced by the cisplatin treatment (Martinot et al., 2018). Cisplatin resistant tumoral cells have an increased ability to repair DNA lesions or a remarkable tolerance to side lesions. As a consequence, the apoptosis pathways are compromised and the cells survive (Florea and Büsselberg, 2011; Galluzzi et al., 2014; Aldossary, 2019).

A widely recognized mechanism of resistance by acquired mutation involves *p53* gene, which can be mutated or compromised due to an increased copy number of MDM2. Nonetheless, in contrast to other cancers, *p53* gene tends to be wild-type in TGCTs, which could explain the overall susceptibility to treatment, yet the mutations that do occur do not account for the greater part of observed chemoresistance (Honecker and Jacobsen, 2015; Romano et al., 2016). In exchange, alterations of apoptosis, autophagy and other intracellular pathways proposed as possible mechanisms for chemoresistance are associated with epigenetic mechanisms, usually entailing expression level changes such as those of DNA repair effectors associated with promoter DNA methylation) in the absence of specific mutations (Alam et al., 2021; Singh et al., 2021a). Pluripotency genes *NANOG* and *OCT3/4* are correlated with the degree of cisplatin sensitivity, however they are associated with epigenetic alterations (Hart et al., 2005; Mueller et al., 2010; Looijenga et al., 2011; Pierpont et al., 2017).

The consequences of cisplatin resistance call for alternative therapies and different directions have been investigated, from other chemotherapy agents such as cabazitaxel, to immunotherapy agents such as avelumab and pembrolizumab, to PARP inhibitors and, most recently, epigenetic drugs, like 5-azacytidine and romidepsin (Adra et al., 2018; Mego et al., 2019; Oing et al., 2019; de Vries et al., 2020). While these medications are under clinical or pre-clinical studies at present, there is evidence to believe that a multimodal approach, which combines them with platinum-based therapy, would bring the most successful outcomes in the future (Martinot et al., 2018). Unfortunately, the fact that the major driver mutations or epigenetic changes are not elucidated, translates into a lack of targeted therapies (Singh et al., 2021b).

Bleomycin’s mechanism of action is still not fully understood and limited information on the genes involved in the sensitivity or resistance to this compound are available (Constantin and Widmann, 2020). Bleomycin seems to selectively inhibit the synthesis of DNA, but at high concentrations, it also suppresses cellular RNA and protein synthesis. *In vitro* studies demonstrate that the DNA-cleaving actions of bleomycin are dependent on oxygen and metal ions, such as iron to produce reactive oxygen species (ROS) involved in DNA cleavage, lipid peroxidation and mitochondrial DNA damage (Dorr, 1992).

The mechanism of resistance to bleomycin can be explained by the presence of a bleomycin hydrolase enzyme that replaces a terminal amino with a hydroxyl group, thereby inhibiting iron binding and cytotoxic activity (Dorr, 1992). An interesting recent study performed by Constantin and Widmann identified ASH2L (Absent, Small, or Homeotic-Like 2), a key component of the H3K4 methyl transferase complex, as a protein required for bleomycin sensitivity (Constantin and Widmann, 2020).

The mechanisms emphasizing the correlation of epigenetic processes with cisplatin and bleomycin sensitivity are further discussed.

### Epigenetic Therapeutic Approach

Epigenetic mechanisms have been associated with different subtypes of TGCTs and with cisplatin resistance (Rijlaarsdam et al., 2015). As previously mentioned, the *OCT3/4* pluripotency gene participates in chemosensitivity. The OCT3/4 protein is associated with p21, whose cellular localization has been associated with resistance to cisplatin (Koster et al., 2010). Specifically, low levels of OCT3/4 protein as well as high levels of cytoplasmic p21 protein have been associated with strong drug resistance, linking the OCT3/4/miR-106b/p21 pathway to drug resistance in TGCTs. Joint with the hypomethylation of *OCT3/4* observed in subtypes of TGCTs, the interplay of multiple epigenetic mechanisms becomes apparent and advocates for a more integrated approach to chemoresistance (Vega et al., 2012; Facchini et al., 2018; Martinot et al., 2018).

DNA methylation has been widely studied, being acknowledged as an important contributor to chemoresistance and chemosensitivity. Cisplatin treatment can induce increased DNA methylation *in vivo* and cisplatin resistance of NSGCT subtypes has been associated with hypermethylation (Bucher-Johannessen et al., 2019). Briefly, global hypermethylation both at CpG and non-CpG loci is associated with cisplatin resistance, while hypomethylation, which usually occurs in SGCTs, is associated with cisplatin sensitivity. DNA methylation at specific gene level is less established. Of the known genes, *RASSF1A* and *CALCA* promoter hypermethylation have been associated with cisplatin resistance (Markulin et al., 2017; Martinelli et al., 2017). Furthermore, a complex interplay between DNA methylation and the polycomb pathway participates in chemoresistance. Studies on cisplatin resistant EC lines have shown that the polycomb pathway is involved in the regulation of cisplatin sensitivity, with polycomb target genes being coregulated by H3K27 methylation and DNA methylation. This could provide yet another epigenetic target, as induction of H3K27 methylation with the demethylase inhibitor GSKJ4 manages to increase cisplatin sensitivity (Fazal et al., 2020; Singh et al., 2021a; Singh et al., 2021b). Another study has shown a decrease in polycomb repressive complex 2 (PRC2) activity in cisplatin refractory cells, together with a global decrease of epigenetic markers H3K27me3 and H2AK119ub, and expression of BMI1 (Singh et al., 2019b).

On the other hand, the epigenetic states of stem cells of TGCT are distinct from those of somatic tumors. Both SGCTs and ECs express OCT3/4, NANOG and other pluripotency proteins in significantly higher levels than somatic cancer stem cells, which is also linked with TGCTs curability. Conversely, cancer stem cells from somatic tumors tend to be resistant to chemotherapy and they express low levels of pluripotency markers (Looijenga et al., 2011; Singh et al., 2021a). Furthermore, one study shows that NANOG and POU5F1 proteins are not expressed in tumors obtained from cisplatin resistant metastases, strengthening their connection with sensitivity and resistance mechanisms (Taylor-Weiner et al., 2016). Given the various epigenetic mechanisms that could explain both sensitivity and resistance, multiple therapeutic targets of epigenetic nature have been investigated, with DNA methyltransferase inhibitors (DNMTi) being the furthest advanced. Demethylating agents and HDACs inhibitors could produce “epigenetic priming of a tumor”, turning it into a more responsive tumor to conventional chemotherapy (Lobo et al., 2021). Studies on EC cell lines have shown a hypersensitivity to DNMTi candidates: decitabine, 5-azacytidine and guadecitabine, compared to somatic tumors. This hypersensitivity is however dependent on high levels of DNMT3b. Treatment of cisplatin refractory cells with DNMTi could re-sensitize them to cisplatin in EC and SGCT (Albany et al., 2017; Albany et al., 2021).

SGCTs and ECs may be sensitive to histone targeting drugs due to their pluripotent nature and the presence of bivalent markers. The effects of such drugs include the activation of the apoptosis cascade, alterations in gene expression and differentiation, and loss of pluripotency. Some candidates are HDAC inhibitors belinostat and panabinosat, which have shown antitumor effects in cisplatin sensitive as well as resistant EC cell lines and xenografts (Lobo et al., 2020a). Another HDAC inhibitor, animacroxam, has shown antitumor effects in cisplatin resistant TGCT cell lines both *in vitro*, and *in vivo* (Steinemann et al., 2019). SGCTs and ECs are also sensitive to the bromodomain inhibitor JQ1 and inhibitors of LSD1, since more undifferentiated TGCT subtypes overexpress LSD1 (Jostes et al., 2019). However, TGCTs subtypes that are more differentiated have a decrease in bivalent histone marks H3K27me3 and H3K4me3 and may benefit less from this line of treatment (Lobo et al., 2020b). Moreover, a recent study suggests the use of a combination of two HDAC inhibitors, suberoylanilide hydroxamic acid (SAHA) and valproic acid as a treatment option for testicular cell carcinoma (Maggisano et al., 2014).

After cisplatin treatment, the remaining cells often differentiate to teratoma, which are resistant to cisplatin treatment, and the treatment usually resorts to surgical resection (Lobo et al., 2020a). From an epigenetic standpoint, this subtype is characterized by a replacement of miR-371a-3p by miR-885-5p, which may contribute to cisplatin resistance (Lobo et al., 2019; Lobo et al., 2020b). A proposed model for cisplatin treatment outcomes is that of “rock and hard place”, which refers to the options of either apoptotic death due to chemosensitivity, or further differentiation under selective pressure. The latter entails acquisition of cisplatin resistance, which leads to loss of tumorigenicity, as the model predicts a link between tumorigenicity and sensitivity to cisplatin. The coupling of the epigenetic states driving both tumorigenicity and chemosensitivity can be lost in rare cases, which pushes for the need of epigenetic drugs that can restore the coupling (Singh et al., 2021b).The persisting effects of cisplatin treatment long after it is completed also need to be taken into consideration. While rat studies have shown a susceptibility to DNA denaturation and strand breaks, after a recovery period, the sperm shows no significant DNA damage. However, epigenetic changes persist, with a protamination level much reduced compared to normal sperm. The effect is explained by an up-regulation of histone variants H1.2, H4, H2A1 and H2B1A (Vega et al., 2012). This type of changes raises concerns for future progeny, as epigenetic alterations are detrimental to proper development and the extent of the consequences of such modifications is unknown.

A study performed by Constantin and Widmann (2020) indicates that patients with low levels of ASH2L or H3K4me3 are more likely to relapse when treated with DNA damaging agent as bleomycin (Constantin and Widmann, 2020). ASH2L is a component of the Set1/Ash2 histone methyltransferase complex, which specifically methylates K4 of histone H3, unless the neighboring K9 residue is already methylated. When ASH2L is downregulated, low H3K4me3 levels have been detected. Recent studies provide evidence that this mark influences the DNA repair ability of cells. A decrease in H3K4me3 mark results in a higher proportion of heterochromatin within nucleus and as a consequence, it is difficult for DNA cleavage agents like bleomycin to access a “close chromatin” conformation and to cause DNA double strand breaks (Constantin and Widmann, 2020). The results presented in this study indicate that patients with low levels of ASH2L or H3K4me3 are more likely to relapse when treated with DNA damaging chemotherapy. The authors indicate that it is possible because the cells with reduced H3K4me3 levels seem to have a selective advantage compared to other cells when it comes to repairing their DNA in response to chemotherapy. In accordance with these results, low levels of ASH2L in cancer cells could be used as a biomarker to predict genotoxin resistance. A very recent study has shown the reciprocal epigenetic modification mediated by DNMT3B and polycomb proteins could be “the key driver” of the cisplatin and DNMTs inhibitors to hypersensitize the TGCTs (Singh et al., 2022). In their research, Singh and collaborators highlighted a crosstalk between H3K27me3 demethylase (KDM6B) and DNMT3B. In this context, they predicted that patients resistant to cisplatin may have high levels of DNMT3B and KDM6B and low levels of H3K27me3. These patients may be hypersensitive to DNMTs inhibitors, and would be also candidates for a combination of epigenetic drugs and cisplatin (Singh et al., 2022).

Another very important area in cancer research is that relating to cellular heterogeneity within a tumor. Within most tumors there are a large number of different cell types other than the cancer cells themselves, including inflammatory and immune cells recruited to the tumor, tissue stroma, vascular endothelial cells, and others. Moreover, the cancer cells of the same type are not genetically identical and exhibit different gene expression patterns. In testicular cancers, but not limited to them, the heterogeneous distribution of distinct tumor cells subpopulations leads to conventional therapeutic strategies predisposing to non-uniform responses, with low healing rate. Some cancer cells subpopulations (drug-sensitive cells) are eliminated, whilst other (drug-resistant cells) remain unharmed. A study performed by Umbreit et al. highlighted the importance of a stem-cell origin of cancer and the cellular context in TGCT patients, with therapeutic implications. An embryonal carcinoma (a chemosensitive progenitor) and a teratoma (a chemoresistant progenitor) have the same genetic defects because of their common origin, but have different epigenetic profiles. The presence of teratoma in the primary tumor seems to have a potential lethal phenotype, resistant to chemotherapy, that may require alternative therapeutic strategies (Umbreit et al., 2020).

Thus, due to their unique germ cell origins, germ cell tumors are associated with distinct epigenetic traits that may be a potential target to treat this malignancy. However, identifying these epigenetic markers and their cross-talk remains a long-standing challenge.

## Conclusion

This review highlights the complexity of germ cells cancer biology, challenging the identification of biomarkers specific to cancer types, cell stages, as well as of those specific to cells’ resistance to conventional chemotherapeutic agents. Unveiling these aspects will allow revolutionary progress in the early diagnosis of cancer and especially in streamlining treatments through combinations of conventional genotoxic and epigenetic therapeutic agents to improve therapeutic outcomes.

The miRNAs, piRNAs and chromatin modifying enzymes show a promising potential as non-invasive cancer biomarkers for clinical applications, such as cancer screening, subtype classification, prognosis and drug sensitivity prediction for treatment strategy selection. The perspective of reversing or modulating epigenetic modifications by specific enzymes inhibitors (DNMTi, HDACi, etc) is encouraging and should motivate future research for the development of novel targeted therapies.

High-throughput methods and bioinformatics tools, mathematical models of cellular systems, alongside nanotechnology will undoubtedly develop further in the coming years leading to the emergence of target-specific epigenetic drugs and the development of precision medicine.
